# Pregnant women’s satisfaction with the quality of antenatal care and the continued willingness to use health facility care in Lusaka district, Zambia

**DOI:** 10.1186/s12884-023-06181-5

**Published:** 2024-01-02

**Authors:** Ladislas Hibusu, Joshua Sumankuuro, Netsai Bianca Gwelo, Olagoke Akintola

**Affiliations:** 1 School of Public Health, Faculty of Community and Health Sciences, University of the Western Cape, Bellville, South Africa; 2 SoCha, LLC, Subdivision 699/Stand 100, Ibex Hill Rd, Lusaka, Zambia; 3 Department of Public Policy and Management, Faculty of Public Policy and Governance, SDD UBIDS, Wa, Ghana; 4 School of Allied Health, Exercise and Sports Sciences, Faculty of Science and Health, Charles Sturt University, Bathurst, NSW Australia

**Keywords:** Maternal health services, Antenatal care, Patient satisfaction, health care utilization, Healthcare acceptability, Zambia

## Abstract

**Background:**

Antenatal healthcare (ANC) reduces maternal and neonatal deaths in low-middle-income countries. Satisfaction with ANC services and perception of quality of care are critical determinants of service utilization. The study aimed to assess pregnant women’s satisfaction with ANC and identify sociodemographic factors associated with satisfaction and their continued willingness to use or recommend the facility to relatives or friends, in Lusaka district, Zambia.

**Methods:**

This was a cross-sectional study involving 499 pregnant women in Lusaka district. A combination of stratified, multistage, and systematic sampling procedures was used in selecting health facilities and pregnant women. This allowed the researcher to assess exposure and status simultaneously among individuals of interest in a population. Structured survey instruments and face-face-interview techniques were used in collecting data among pregnant women who were receiving ANC in selected health facilities.

**Results:**

Overall, the proportion of pregnant women who were fully satisfied with ANC was 58.9% (*n* = 292). Pregnant women’s satisfaction score ranged from physical aspects (40.9 - 58.3%), interpersonal aspects (54.3 - 57.9%) to technical aspects of care (46.9 - 58.7%). Husbands’ employment status (OR = 0.611, 95%CI = 0.413 – 0.903, *p* = 0.013), monthly household income level of > 3000 - ≤6000 Kwacha (OR = 0.480, 95%CI = 0.243 – 0.948, *p* = 0.035 were significantly associated with the interpersonal aspects and the physical aspects of care, respectively. Besides, pregnant women who were in their third trimester (above 33 weeks), significantly predicted satisfaction with the physical environment of antenatal care (OR = 3.932, 95%CI = 1.349 – 11.466, *p* = 0.012). In terms of the type of health facility, women who utilized ANC from Mtendere (OR = 0.236, 95% CI = 0.093 – 0.595, *p* = 0.002) and N’gombe (OR = 0.179, 95% CI = 0.064 – 0.504, *p* = 0.001) clinics were less satisfied with the physical environment of care. Place of residence and educational attainment showed significant association with ‘willingness to return‘. N’gombe clinic (*n* = 48, 77.4%) received the lowest consideration for ‘future care’.

**Conclusion:**

Drawing on Donabedian framework on assessing quality of healthcare, we posit that pregnant women’s satisfaction with the quality of antenatal care was low due to concerns about the physical environment of health facilities, the interpersonal relationships between providers and pregnant women as well as the technical aspects of care. All these accounted for pregnant women’s dissatisfaction with the quality of care, and the indication of unwillingness to return or recommend the health facilities to colleagues. Consistent with Donabedian framework, we suggest that the codes and ethics of healthcare must be upheld. We also call for policy initiatives to reshape the physical condition of ANC clinics and to reinforce healthcare providers’ focus on the ‘structures’ and the ‘processes’ relevant to care in addition to the ‘outcomes’.

**Supplementary Information:**

The online version contains supplementary material available at 10.1186/s12884-023-06181-5.

## Introduction

Maternal mortality remains a public health problem in low and middle income countries [1, 2]. Indeed, WHO found that health complications such as bleeding during pregnancy or childbirth, high blood pressure, obstructed labour, HIV, malaria, tuberculosis and puerperal sepsis account for a quarter of all maternal mortalities in sub-Saharan Africa [2]. However, the literature shows that many of these morbidities which could result in mortalities are preventable through quality care during pregnancy and childbirth [3, 4]. Thus, WHO recommends quality antenatal care (ANC) as an effective programme, that aim to reduce avoidable maternal morbidities and mortalities [3]. ANC is an umbrella term used to describe the care that is provided to pregnant women throughout pregnancy until the child’s birth and is aimed at detecting existing problems and those that could develop during pregnancy, to affect the pregnant woman and/or her unborn child [4]. ANC covers various screening tests, diagnostic procedures and prophylactic treatments, some of which are done routinely, and others provided to the women based on identified problems and risk factors [4].

In addition, antenatal care focuses on increasing ‘access to care’ and ‘quality of care’[3]. Levesque et al. [5] define ‘access to care’ as the approachability, acceptability, availability, affordability, and appropriateness of antenatal care (ANC). Antenatal care utilisation in most low-and middle-income countries is determined by both ‘supply-side’ (i.e., healthcare providers, staff training, and medical supplies) and ‘demand-side’ (sociodemographic characteristics, physical distance to care location, previous experiences at health facilities, medical screening, culture, etc.) factors [6]. In the context of this study, one could surmise that pregnant women’s ability to utilise ANC services that are acceptable, affordable and satisfactory, will enhance positive health and pregnancy outcomes. The WHO [2] defines ‘quality of care’ as the degree to which health services for individuals and populations increase the likelihood of desired health outcomes. Sheffel et al. [7] and Galle et al. [8] state that the quality of antenatal care and experience of care improves health outcomes, determines return visits (continuity of care), treatment adherence, and improves the relationship with the providers.

Therefore, over the years, studies have shown that multiple factors have prevented increased utilisation of ANC services [9–14]. Specifically, ANC settings, health professionals’ attitudes towards pregnant women when they seek care, and logistical problems at the health facility levels, among others have accounted for low satisfaction with care and low ANC utilisation in low-middle-income [11, 13–20]. Notably, the measurement of quality of care has historically focused on the availability of healthcare workers and the provision of basic services. The literature shows the physical environment of ANC, provider ethical conduct and interpersonal relationship between patients and providers determine patient satisfaction [8, 10, 12, 21]. These necessitated increased calls for a focus on the quality of ANC and patient satisfaction [22].

Besides these factors, patient satisfaction is a reflection of the patient’s judgment of the extent to which they are satisfied with their experience of care in different domains of health care including technical, interpersonal, and organizational aspects [12, 23]. Satisfaction with the quality of ANC received and patient willingness to return or recommend the facility to other pregnant women in low and middle-income countries remain critical factors in addressing maternal mortality.

In this study willingness defines the satisfaction pregnant women receive during ANC. First, pregnant women seek care with the expectation of the treatment and outcome, how they feel about the care provided, and their perception of it. Second, pregnant women have many choices for healthcare, and their experience. Thus, client loyalty is somewhat influenced by their perception and experiences about care. In a systematic review in sub-Saharan Africa, Moyer et al. [18] noted that pregnant women’s experiences during care influence loyalty to the health facility, their decisions and their willingness to return or recommend the same to others [18]. Katemba et al. [10] reported that 52.9% of pregnant women attending ANC in Zambia received low-quality care. From the discussion so far, the evidence suggests that pregnant women’s satisfaction with the quality of ANC will motivate them to repeat the visit and use care in the same facility in the future as well as recommend the same facility to other pregnant women.

**Fig. 1:**
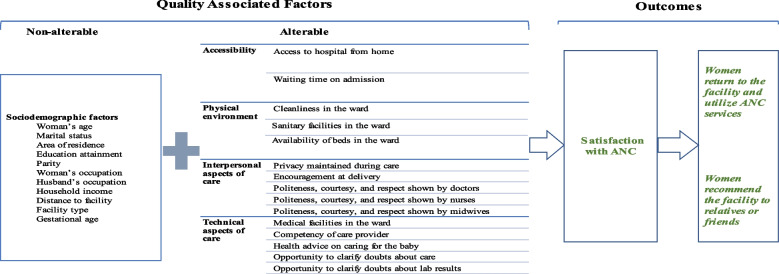
Factors that influence satisfaction with ANC. Adapted from Donabedian framework [26]

Donabedian’s conceptual framework on ‘quality of healthcare’ [24], is a useful tool for examining pregnant women’s satisfaction with the quality of ANC and their willingness to return or recommend their ANC health facilities to relatives and friends. Donabedian argues that three broad dimensions influence the quality of healthcare, i.e., *structure, process, and outcome. Structure* denotes the attributes of the settings in which care occurs including the physical environment. *Process* denotes what is done in providing and receiving care (interpersonal and technical aspects of care) while *outcome* denotes the effects of care on the health status of patients and populations [25]. The author further argues that the three-part approach to quality assessment is possible only because good structure increases the likelihood of a good process, and a good process increases the likelihood of a good outcome (see Fig. 1). In applying this conceptual framework to our study, we hope to illuminate the factors associated with pregnant women’s satisfaction with ANC and their willingness to return to an ANC facility.

The literature suggests that quality of care in a given health facility is an important precondition for pregnant women’s satisfaction with ANC as well as the continued use of that facility care and repeated visits. Many researchers believe that satisfied patients are more likely to return or recommend a particular ANC clinic to others [8, 10, 15–17, 21, 27, 28]. While there is extensive research evidence on ANC uptake and ANC quality in the Lusaka district, Zambia [10], we know very little about pregnant women’s satisfaction with ANC and their willingness to return or recommend the same facility to relatives and friends. This paper examines pregnant women’s satisfaction with the quality of antenatal healthcare received at health facilities and their willingness to return to the same facilities or recommend them to relatives or friends in Lusaka, Zambia.

## Methods

### Research setting

This research was conducted in Lusaka district, which is situated in Lusaka province, Zambia. The district encompasses, all levels of health services from primary health facilities (health posts) to a tertiary facility (a University Teaching Hospital). Lusaka is one of the smallest and yet one of the most populated and highly urbanised provinces in Zambia with an estimated total population of about 2.8 million people [29]. The estimated population of women of childbearing age (WCBA) in Lusaka district was 735,361 with approximately 100,919 expected pregnancies and about 100,545 expected deliveries in 2022 [29].

### Study population

Study participants were all pregnant women attending ANC at any stage of their pregnancy (first, second, third or fourth trimester) within Lusaka district. The following inclusion and exclusion criteria were applied:

### Inclusion criteria

The study included pregnant women who were attending ANC at the sampled health facilities. Pregnant women were also included in the sample if they:

- were 18 years old and above;
- could speak a known/common local language (such as Nyanja) in the areas where the study sites are located.
- could give informed consent.

### Exclusion criteria

The study excluded:

- pregnant women who were attending emergency ANC on the day of potential recruitment for ethical reasons and the potential impact on health outcomes.
- pregnant women who were acquainted with the study research assistants (to respect confidentiality and avoid bias).
- pregnant women that were less than 18 years old for ethical reasons.

### Sampling and sample size determination

Lusaka district has 59 documented public health facilities clustered in six sub-districts or Zones: Chelstone, Chipata, Matero, Kanayama, Chawama, and Chilenje [16]. Of this number, 39 public health facilities provide primary health care [10]. ANC is provided in primary healthcare facilities in the study district. In selecting the facilities for inclusion in the study, a multi-stage sampling technique was used [30]. First, a random sampling of eight health facilities that offer antenatal care from the 39 identified facilities was conducted [16].

Second, we applied Cochran formula for sample size calculation, which was reported in a previous study in Zambia [16]:

$$n={Z}^2(deff)p/{e}^2$$

Where n = the desired sample size.

Z^2^ (standard normal deviate at 95% confidence level) = 1.96.

P = (proportion of overall client satisfaction with antenatal services from the previous study in Lusaka found to be 0.29 or 29% )[10];

e = measure of precision. In this study, the margin of error was set at 0.05,

Deff = is the design effect set at 1.5. The estimated sample size therefore was:

$$\textrm{n}={1.96}^{2\ast }0.29(1.5)\left(1-0.29\right)/{0.05}^2=475.$$

To account for withdrawal and non-responses from the study, the sample size was adjusted as follows: Where *nf* is the final sample size, r is the response rate in decimals which was found to be 95.8% (0.958) for the 2018 Zambia Demographic Health Survey.

$$\textrm{nf}={}^{\textrm{n}}\!\left/ \!{}_{\textrm{r}}\right.;\textrm{therefore},\textrm{nf}={}^{475}\!\left/ \!{}_{0.958}\right.=495$$

Thus, the required total sample size for the study was 495.

**Fig. 2:**
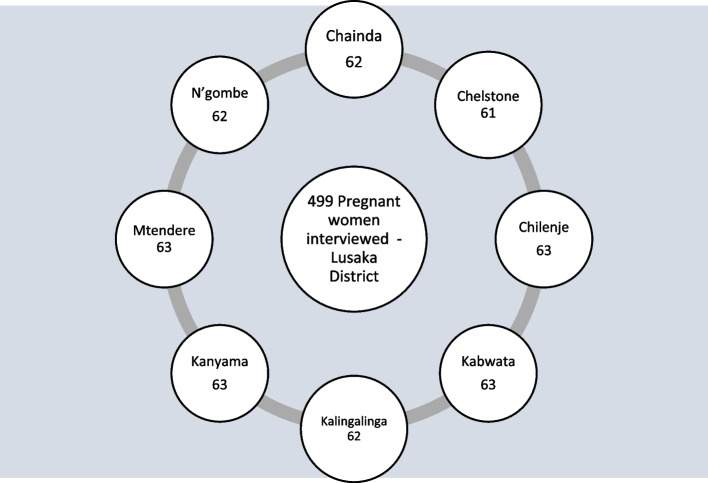
Sampled health facilities and number of pregnant women interviewed

Systematic sampling procedure was then used to select the participants from the eight health facilities for the study. The distribution of the final sampled participants is presented in Fig. 2.

### Instrumentation

We adopted the WHO pretested questionnaire which was used by Senarath, Fernando, Rodrigo for a similar study in Sri Lanka [31]. The instrument was translated into ‘Nyanja’, which was the most spoken local language in Lusaka district. The questionnaire used for the data collection was divided into various sections: the socio-demographic characteristics, rating of antenatal care services; willingness to come back to the same facility if they are pregnant again and if they would recommend the facility to a relative or a friend. Satisfaction was assessed using five-point response categories: (fully dissatisfied, somewhat dissatisfied, neither satisfied nor dissatisfied, somewhat satisfied, fully satisfied) with ‘1’ being fully dissatisfied and ‘5’ being very satisfied [13, 31].

### Data collection

Structured quantitative data collection instruments were used to collect data from pregnant women who were attending the ANC clinic. Research assistants were trained on data collection procedures, question interpretation, usage of mobile devices for data collection, COVID-19 protocols/guidelines and ethics in research, before data collection. Mobile devices were programmed and used in collecting the data. However, all data were collected using a face-to-face interview technique. Data was collected as exit interviews at convenient venues (private spaces) within the health facility settings. Completion of each questionnaire lasted between 30 to 45 minutes. Data was collected in the local language, ‘Nyanja’.

### Participant recruitment

The study was conducted by the first author with the support of field assistants. First, the first author and the field assistants went around all the selected antenatal care facilities to present the ethics approval and MOH documentation to the health facility managers who are also known as facility ‘In-Charge’. Second, the participant information sheet which contained a detailed explanation of the research aims and the use of the research data was distributed to those who could read in English or *Nyanja.* Verbal explanations were given to those who could not read in any of these languages. Finally, participants were given the chance to obtain explanations concerning the study, before deciding to participate.

### Outcome variable

This study measured three outcomes. The primary outcome variable was to determine the proportion of pregnant women who are fully satisfied with the ANC that they receive from the health facilities they visit. The secondary outcomes were two-fold; to determine whether satisfaction was directly related to women’s intention to use the same facility in future if they fall pregnant again, and to determine if satisfaction influences the willingness of women to recommend the facility to relatives and friends. We measured satisfaction with ANC using a 16-item instrument adapted from a previous study [32]. The items covered several key dimensions of client satisfaction: accessibility (two questions), interpersonal aspect of care (five questions), physical environment (three questions), technical aspects of care (four questions) and outcome of care (two questions). The responses were marked using a 5-point Likert-type scale [32]: (1) fully satisfied, (2) somewhat satisfied, (3) neither satisfied nor dissatisfied, (4) somewhat dissatisfied and (5) fully dissatisfied. Because of the known limited validity of questions that include the word “satisfaction”, the study adapted one direct question and two indirect questions from a previous antenatal care trial [28]. The direct question read: *to what extent are you satisfied with the antenatal care you have received in this clinic so far?).* The two indirect questions were also asked to provide further understanding: *would you come back to this clinic; and would you recommend this clinic to a relative or friend*? The general satisfaction question/variable aimed to synthesize women’s overall perception of the quality of ANC (see Supplementary file 1).

### Explanatory variable

Explanatory variables are the sociodemographic characteristics of the pregnant women that participated in the study. These are; the participant’s age; marital status; area of residence (whether the woman resided in a low, medium or high-density area); education attainment; parity (number of children); the employment status of the women; the employment status of the husband; household monthly income; time it took to reach the health facility (used to estimate the distance to the health facility); level of clinic/hospital; and gestational age (reported in weeks).

### Threats to validity and reliability

We employed three key approaches to ensure the data collected and the analysis, were valid and reliable. First, we managed potential selection bias of the instruments by adapting a pre-tested instrument that was designed by the World Health Organization and used in an earlier study [31]. In that study, the instrument reported a high internal consistency (Cronbach’s α = 0.81). Additionally, to ensure the representation of study participants, health facilities were randomly selected, and participants were selected using a systematic sampling procedure. This further minimized bias especially because we ensured careful identification, and selection of respondents. Finally, we recognise the effect of the time differences between the experiences and perceptions of women in the first trimester and those advanced in gestational age. Differences might also be observed between first-time pregnancies and those who had been pregnant before and accessed ANC. These biases were minimized at the analysis stage by comparing experiences by stage of pregnancy.

### Data processing and analysis

First, the questionnaire forms were checked for completeness at the close of each interview. Completed data was then exported to STATA (version 13) data analysis software, entries checked and cleaned and then frequencies and percentages for the variables were calculated to identify patterns and those with missing values.

Second, bivariate analysis was conducted on the sociodemographic variables and maternal characteristics (independent variable) and ‘willingness to return’ (dependent variable) to the health facility if a woman falls pregnant. We also conducted bivariate analysis for the sociodemographic variables and ‘willingness to recommend’ the health facility to friends and relatives to estimate the odd ratios for the variables. Finally, sociodemographic variables with *p* < 0.05 were retained in the multiple logistic regression model to identify the specific aspects of the independent variables that had associations with ‘client satisfaction’ and relevant sociodemographic and ANC-related characteristics. To determine the factors associated with client satisfaction, three composite outcome variables were constructed, i.e., interpersonal aspects of care, technical aspects of care and physical environment. Satisfaction with interpersonal aspects was defined as the proportion of respondents who reported ‘fully satisfied’, or ‘not fully satisfied’ [31]. Similarly, satisfaction with ‘technical aspects’ and the ‘physical environment’ was derived from the respective variables. During logistic regression modelling, not ‘fully satisfied’ were combined to encompass all other satisfaction levels other than ‘fully satisfied’ i.e., somewhat satisfied, neither satisfied nor dissatisfied, somewhat dissatisfied and fully dissatisfied. Statistical analysis was conducted at a 95% confidence level, and statistical significance was considered at *p* < 0.05.

## Results

### Participant characteristics

Table 1 shows the relevant sociodemographic and healthcare-related characteristics of pregnant women. Of the 499 pregnant women interviewed, 42.7% (*n* = 213) were below 25 years with the mean age estimated at 26 years. A large proportion of 33.7% (*n* = 168) were between the ages of 20-24 years. The majority, 72.3% (*n* = 361) were married while 27.7% (*n* = 138) were either single, divorced, living with a stable partner, or widowed. Most of the women, 63.1% (*n* = 315) resided in high-density places (crowded settlements), while more than half of the respondents, 52.9% (*n* = 264) had attained a secondary level of education, More than half, 71.3% (*n* = 356) already had a child or more. Employment status among pregnant women was relatively low (n = 138, 27.7%) while more than half of their spouses/husbands were employed (*n* = 266, 53.3%). Most households, 78.6% (*n* = 392), earned a monthly income of less than three thousand Kwacha (≤ ZWM 3000), and the majority took less than 30 minutes to reach a health facility (n = 361, 72.3%). In this study, the mean time to reach the health facility by the majority of women was 28.3 (SD = 22.7).

**Table 1:** Sociodemographic characteristics of participants (*n* = 499)

Variable	n	%
**Maternal age**		
<20	45	9.0
20 – 24	168	33.7
25 – 29	146	29.3
30 – 34	91	18.2
35 – 39	37	7.4
≥40	12	2.4
**Mean age**	**26.3**	**SD = 5.7**
**Marital status**		
Single	138	27.7
Married	361	72.3
**Area of residence**		
Low density	36	7.2
Medium density	148	29.7
High density	315	63.1
**Education attainment**		
None	14	2.8
Primary	165	33.1
Secondary	264	52.9
Tertiary	56	11.2
**Parity (no. children)**		
Primiparae	143	28.7
Multiparous	356	71.3
**Mean parity**	**1.5**	**SD = 1.4**
**Employment of pregnant women**		
Employed	138	27.7
Not employed	361	72.3
Spouse/husband’s occupation*		
Employed	266	53.3
Not employed	233	46.7
**HH income (Kwacha)**		
≤3000	392	78.6
>3001 - ≤6000	73	14.6
>6000	34	6.8
**Mean HH income**	**28.3**	**SD = 22.7**
**Time to hospital**		
<30 mins	361	72.3
≥30 mins	138	27.7
**Mean time to clinic**	**28.3**	**SD = 22.7**
**Level of hospital**		
First Level Hospital	126	25.3
Urban Health Centre	373	74.7
**Pregnancy (weeks)**		
<12 weeks	44	8.8
13 – 26	243	48.7
27 – 32	181	36.3
Above 33 weeks	31	6.2

### Client satisfaction

Table 2 presents the proportion of pregnant women who were satisfied with each of the 16 items and the mean satisfaction score for each item. The 16 items were grouped into five categories: general, accessibility, interpersonal, technical, and physical environment aspects. The satisfaction score was constructed by giving a graded score; where fully dissatisfied was assigned to 1 and 5 was assigned to fully satisfied.

**Table 2:** Level of satisfaction with antenatal care (*n* = 499)

	Fully satisfied %(n)	Somewhat satisfied %(n)	Neither satisfied nor dissatisfied %(n)	Somewhat dissatisfied %(n)	Fully dissatisfied %(n)	Mean satisfaction
**General**						
General satisfaction with ANC services received	58.5 (292)	8.4 (42)	32.3 (161)	0.8 (4)		4.25
**Accessibility**						
Access to hospital from residence	60.9 (304)	7.0 (35)	29.9 (149)	2.2 (11)		4.27
Waiting time on admission	55.9 (279)	13.4 (67)	28.3 (141)	2.4 (12)		4.23
**Interpersonal aspects of care**						
Privacy maintained during the care	57.9 (289)	10.6 (53)	30.7 (153)	0.8 (4)		4.26
Encouragement at delivery	56.9 (284)	10.4 (52)	31.9 (159)	0.8 (4)		4.23
Politeness, courtesy, and respect shown by doctors	56.1 (280)	12.0 (60)	31.1 (155)	0.8 (4)		4.23
Politeness, courtesy, and respect shown by nurses	56.1 (280)	11.8 (59)	31.1 (155)	0.8 (4)	0.2 (1)	4.23
Politeness, courtesy, and respect shown by midwives	54.3 (271)	13.4 (67)	30.7 (153)	1.4 (7)	0.2 (1)	4.20
**Technical aspects of care**						
Medical facilities in the ward	46.9 (234)	14.6 (73)	35.9 (179)	2.2 (11)	0.4 (2)	4.05
Competency of care provider	58.3 (291)	7.2 (36)	33.9 (169)	0.6 (3)		4.23
Health advice on caring the newborn	56.9 (284)	8.0 (40)	33.9 (169)	1.2 (6)		4.21
Opportunity to clarify doubts about care	57.7 (288)	6.6 (33)	35.1 (175)	0.6 (3)		4.21
Opportunity to clarify doubts about lab results	58.7 (293)	6.2 (31)	34.3 (171)	0.8 (4)		4.23
**Physical environment**						
Cleanliness in the ward	58.3 (291)	8.4 (42)	31.3 (156)	2.0 (10)		4.23
Sanitary facilities in the ward	40.9 (204)	10.8 (54)	41.3 (206)	3.6 (18)	3.4 (17)	3.82
Availability of beds in the ward	46.9 (234)	12.2 (61)	39.3 (196)	1.6 (8)		4.04

Satisfaction scores were constructed by giving scores in the following manner: fully satisfied = 5; somewhat satisfied = 4; neither satisfied nor dissatisfied = 3; somewhat dissatisfied = 2; fully dissatisfied = 1

Results show that more than half of the respondents (58.5%) were generally ‘fully satisfied’ with the antenatal care they received from the health facilities. More than 55% of the respondents reported that they were ‘fully satisfied’ with the accessibility to care, i.e., access to the hospital from residence (60.9%) and the waiting time on admission (55.9%).

The proportion of women who were ‘fully satisfied’ with the ‘interpersonal aspects’ of ANC ranged from 54.3 to 57.9% with ‘politeness, courtesy and respect’ shown by midwives (54.3%), receiving the lowest rating in this category. The highest rating was on ‘maintenance of privacy during care’ (57.9%). Under the ‘technical aspects’ of ANC, the ‘availability of medical facilities in the ward’ was rated low (46.9%) in this category with the ‘opportunity to clarify doubts about laboratory result’ (58.7%) receiving the highest satisfaction rating in the category. Satisfaction with the ‘physical environment’ in the wards was low when compared with other aspects of care; only 40.9% were fully satisfied with the sanitary facilities and 46.9% with the availability of beds.

### Sociodemographic characteristics and ANC satisfaction

Five variables on patient and healthcare-related characteristics were significant factors in at least one satisfaction dimension in the bivariate analysis. The husband’s employment, household income (Kwacha), time to the hospital, gestational age, and the health facility (clinic) were significantly associated with satisfaction with ANC across different dimensions (*p* < 0.05). One factor (gestational age) demonstrated a consistent significant association across all three dimensions of care (interpersonal, technical and physical environment) (Table 3).

**Table 3:** Factors significantly associated with pregnant women’s satisfaction: logistic regression analysis (*n* = 499)

Characteristics	Interpersonal aspect OR (95% CI) ***p***-value	Technical aspect OR (95% CI) p-value	Physical environment OR (95% CI) p-value
**Spouse/husband’s occupation**			
Employed	1.000	1.000	1.000
Not employed	0.611 (0.413 - 0.903) * 0.013	0.743 (0.499 - 1.105) 0.142	0.878 (0.562 - 1.367) 0.562
**Household Income (Kwacha)**			
≤3000	1.000	1.000	1.000
>3001 - ≤6000	0.655 (0.372 - 1.153) 0.143	0.663 (0.370 - 1.186) 0.166	0.480 (0.243 - 0.948) * 0.035
>6000	2.063 (0.912 - 4.668) 0.082	1.822 (0.833 - 3.987) 0.133	1.880 (0.797 - 4.435) 0.149
**Time to hospital**			
≤30 mins	1.000	1.000	1.000
>30 mins	1.441 (0.944 - 2.199) 0.090	1.557 (1.017 - 2.384) * 0.041	1.771 (1.112 - 2.819) * 0.016
**Gestational (weeks)**			
<12 weeks	1.000	1.000	1.000
13 – 26	0.583 (0.294 - 1.158) 0.123	0.609 (0.302 - 1.232) 0.168	0.553 (0.253 - 1.208) 0.137
27 – 32	1.235 (0.604 - 2.523) 0.563	1.388 (0.673 - 2.863) 0.375	1.887 (0.855 - 4.169) 0.116
≥33 weeks	1.438 (0.541 - 3.826) 0.466	1.841 (0.690 - 4.912) 0.223	3.932 (1.349 - 11.466) * 0.012
**Clinics**			
Chainda	1.000	1.000	1.000
Chelstone	0.959 (0.452 - 2.033) 0.913	1.345 (0.624 - 2.898) 0.449	1.641 (0.748 - 3.601) 0.217
Chilenje	1.300 (0.610 - 2.772) 0.497	1.260 (0.577 - 2.755) 0.562	0.495 (0.210 - 1.168) 0.108
Kabwata	1.527 (0.699 - 3.333) 0.288	2.137 (0.965 - 4.735) 0.061	1.349 (0.592 - 3.073) 0.477
Kalingalinga	1.044 (0.495 - 2.199) 0.911	0.926 (0.427 - 2.089) 0.845	0.836 (0.380 - 1.840) 0.657
Kanyama	0.686 (0.320 - 1.469) 0.332	1.306 (0.603 - 2.828) 0.498	0.858 (0.386 - 1.908) 0.707
Mtendere	0.671 (0.317 - 1.421) 0.297	1.113 (0.518 - 2.394) 0.783	0.236 (0.933 - 0.595) 0.002**
N’gombe	1.151 (0.548 - 2.416) 0.711	1.122 (0.517 - 2.434) 0.771	0.179 (0.064 - 0.504) 0.001**

Pregnant women who were in their third trimester (above 33 weeks), statistically significantly predicted satisfaction with the physical environmental aspects of antenatal care (OR = 3.932, 95%CI = 1.349 – 11.466, p = 0.012)

### Factors associated with ANC satisfaction

Multiple logistic regression results show that women who were married to spouses/husbands, which were not employed predicted lesser satisfaction on interpersonal aspects of ANC care. Thus, women whose husbands were unemployed (OR = 0.611, 95%CI = 0.413 – 0.903, *p* = 0.013), were 0.611 times less likely to be satisfied with the interpersonal aspects of antenatal care (Table 3).

Pregnant women from households that earned > 3000 - ≤6000 significantly predicted satisfaction on the physical aspects of care (OR = 0.480, 95%CI = 0.243 – 0.948, *p* = 0.035), such that these women were about 0.5 times less likely to be satisfied with ANC when compared with women that earned ≤3000 Kwacha (Table 3).

On the time women took to reach the health facility, pregnant women who took more than 30 minutes to reach the health facility significantly predicted satisfaction on two aspects of antenatal care: technical (OR = 1.557, 95%CI = 1.017 – 2.384, *p* = 0.041) and physical environmental (OR = 1.771, 95%CI = 1.112 – 2.819, *p* = 0.016) (Table 3).

Regression analysis further shows that pregnant women whose pregnancy was above 27 weeks (gestational age) predicted higher satisfaction across all dimensions of care when compared to women whose pregnancy was below 26 weeks (first trimester) (Table 3).

**Fig. 3:**
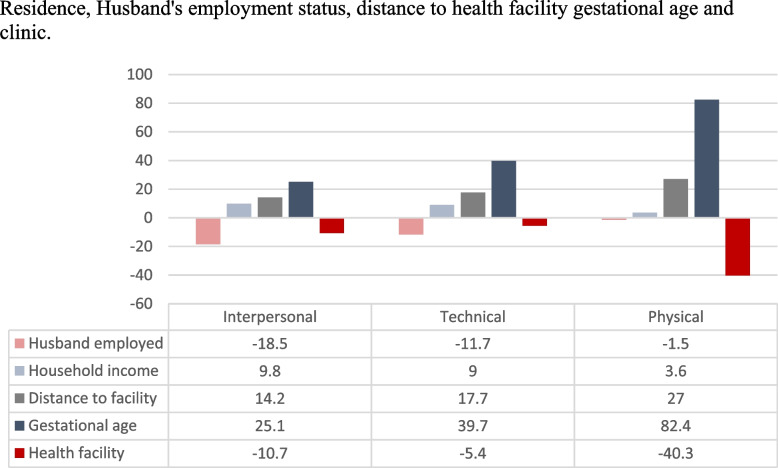
Percentage change in the odds of satisfaction with ANC

The regression analysis also shows that the type of health facility (in this case clinic) was a predictor of antenatal care satisfaction by pregnant women. Mtendere (OR = 0.236, 95% CI = 0.093 – 0.595, *p* = 0.002) and N’gombe (OR = 0.179, 95% CI = 0.064 – 0.504, *p* = 0.001) clinics showed significant association with the physical environmental aspects of antenatal care. When compared to Chainda (a lower-level clinic) to others included in this study – an urban centre, women who sought antenatal care from Mtendere and N’gombe clinics were about 0.2 times less likely to be satisfied with the physical environmental aspects of care. This is further clarified when a 1-standard deviation percentage change was plotted in a bar graph (Fig. 3). The bar graph shows higher dissatisfaction with the health facility (clinic)‘s physical environmental aspects of care negative 40.3% when compared to interpersonal (negative 10.7%) and technical aspects of care (negative 4.7%).

### Willingness to return or to recommend a health facility

Table 4 presents findings on pregnant women’s willingness to return to the health facility if they fall pregnant again or their willingness to recommend a given health facility to a relative or a friend.

**Table 4:** Willingness to return or to recommend a health facility (*n* = 499)

	Willing to Return				Willing to Recommend			
Characteristics	*Yes*	*Maybe*	*No*	*p-value*	*Yes*	*Maybe*	*No*	*p-value*
**Clinic**	n(%)	n(%)	n(%)		n(%)	n(%)	n(%)	
Chainda	55 (88.7)	2 (3.2)	5 (8.1)	0.113	59 (95.2)	1 (1.6)	2 (3.2)	0.000***
Chelstone	56 (91.8)	2 (3.3)	3 (4.9)		52 (85.2)	7 (11.5)	2 (3.3)	
Chilenje	56 (88.9)	4 (6.3)	3 (4.8)		54 (88.5)	5 (8.2)	2 (3.3)	
Kabwata	58 (92.1)	5 (7.9)	0 (0)		57 (95.0)	2 (3.3)	1 (1.7)	
Kalingalinga	57 (91.9)	2 (3.2)	3 (4.8)		60 (96.8)	2 (3.2)	0 (0)	
Kanyama	57 (90.5)	4 (6.3)	2 (3.2)		59 (96.7)	1 (1.6)	1 (1.6)	
Mtendere	57 (90.5)	2 (3.2)	4 (6.3)		57 (90.5)	3 (4.8)	3 (4.8)	
N’gombe	48 (77.4)	4 (6.5)	10 (16.1)		44 (71.0)	11 (17.7)	7 (11.3)	
**Total**	**444 (89.0)**	**25 (5.0)**	**30 (6.0)**		**442 (89.8)**	**32 (6.5)**	**18 (3.7)**	
**Women’s age**	n(%)	n(%)	n(%)		n(%)	n(%)		
Below 20	42 (93.3)	1 (2.2)	2 (4.4)	0.751	42 (93.3)	2 (4.4)	1 (2.2)	0.874
20 – 24	145 (86.3)	11 (6.6)	12 (7.1)		146 (89.6)	12 (7.4)	5 (3.1)	
25 – 29	133 (91.1)	6 (4.1)	7 (4.8)		127 (88.2)	12 (8.3)	5 (3.5)	
30 – 34	82 (90.1)	4 (4.4)	5 (5.5)		81 (89.0)	5 (5.5)	5 (5.5)	
35 – 39	32 (86.5)	3 (8.1)	2 (5.4)		35 (94.6)	1 (2.7)	1 (2.7)	
40+	10 (83.3)	0 (0.0)	3 (16.7)		11 (91.7)	0 (0.0)	1 (8.3)	
**Total**	**444 (89.0)**	**25 (5.0)**	**30 (6.0)**		**442 (89.8)**	**32 (6.5)**	**18 (3.7)**	
**Marital status**								
Single	121 (87.7)	8 (5.8)	9 (6.5)	0.838	120 (90.2)	8 (6.0)	5 (3.8)	0.963
Married	323 (89.5)	17 (4.7)	21 (5.8)		322 (89.7)	24 (6.7)	13 (3.6)	
**Total**	**444 (89.0)**	**25 (5.0)**	**30 (6.0)**		**442 (89.8)**	**32 (6.5)**	**18 (3.7)**	
**Area of residence**								
Low density	29 (80.6)	1 (2.8)	6 (16.7)	0.018*	32 (88.9)	2 (5.6)	2 (5.6)	0.563
Medium density	129 (87.2)	12 (8.1)	7 (4.7)		124 (86.7)	13 (9.1)	6 (4.2)	
High density	286 (90.8)	12 (3.8)	17 (5.4)		286 (91.4)	17 (5.4)	10 (3.2)	
**Total**	**444 (89.0)**	**25 (5.0)**	**30 (6.0)**		**442 (89.8)**	**32 (6.5)**	**18 (3.7)**	
**Women’s educational attainment**								
No formal education	14 (100)	0 (0.0)	0 (0.0)	0.007*	14 (100.0)	0 (0.0)	0 (0.0)	0.172
Primary	148 (89.7)	5 (3.0)	12 (7.3)		147 (90.7)	9 (5.6)	6 (3.7)	
Secondary	240 (90.9)	12 (4.6)	12 (4.6)		238 (90.8)	15 (5.7)	9 (3.4)	
Tertiary	42 (75.0)	8 (14.3)	6 (10.7)		43 (79.6)	8 (14.8)	3 (5.6)	
**Total**	**444 (89.0)**	**25 (5.0)**	**30 (6.0)**		**442 (89.8)**	**32 (6.5)**	**18 (3.7)**	
**Parity (no. children)**								
Primiparae	125 (87.4)	8 (5.6)	10 (6.7)	0.356	125 (89.9)	9 (6.5)	5 (3.6)	0.999
Multiparous	319 (89.6)	17 (4.8)	20 (5.6)		317 (89.8)	23 (6.5)	13 (3.7)	
**Total**	**444 (89.0)**	**25 (5.0)**	**30 (6.0)**		**442 (89.8)**	**32 (6.5)**	**18 (3.7)**	
**Employment of pregnant women**								
Employed	72 (83.7)	7 (8.1)	7 (8.1)	0.208	75 (89.3)	7 (8.3)	2 (2.4)	0.614
Not employed	372 (90.1)	18 (4.4)	23 (5.6)		367 (90.0)	25 (6.1)	16 (3.9)	
**Total**	**444 (89.0)**	**25 (5.0)**	**30 (6.0)**		**442 (89.8)**	**32 (6.5)**	**18 (3.7)**	
**Husband’s employment**								
Employed	236 (88.7)	15 (5.6)	15 (5.6)	0.746	239 (90.5)	17 (6.4)	8 (3.0)	0.091
Not employed	208 (89.3)	10 (4.3)	15 (6.4)		203 (89.0)	15 (6.6)	10 (4.4)	
**Total**	**444 (89.0)**	**25 (5.0)**	**30 (6.0)**		**442 (89.8)**	**32 (6.5)**	**18 (3.7)**	
**Household income (Kwacha)**								
≤ 3000	348 (88.8)	18 (4.6)	26 (6.6)	0.749	346 (89.4)	24 (6.2)	17 (4.4)	0.324
> 3001 - ≤ 6000	65 (89.0)	5 (6.9)	3 (4.1)		65 (89.0)	7 (9.6)	1 (1.4)	
> 6000	31 (91.2)	2 (5.9)	1 (2.9)		31 (96.9)	1 (3.1)	0 (0.0)	
**Total**	**444 (89.0)**	**25 (5.0)**	**30 (6.0)**		**442 (89.8)**	**32 (6.5)**	**18 (3.7)**	
**Time to hospital**								
≤ 30 mins	327 (90.6)	18 (5.0)	16 (4.4)	0.055	324 (91.3)	20 (5.6)	11 (3.1)	0.238
> 30 mins	117 (84.8)	7 (5.1)	14 (10.1)		118 (86.1)	12 (8.8)	7 (5.1)	
**Total**	**444 (89.0)**	**25 (5.0)**	**30 (6.0)**		**442 (89.8)**	**32 (6.5)**	**18 (3.7)**	
**Level of hospital**								
First Level Hospital	113 (89.7)	8 (6.4)	5 (4.0)	0.410	113 (92.6)	6 (4.9)	3 (2.5)	0.495
Urban Health Centre	331 (88.7)	17 (4.6)	25 (6.7)		329 (88.9)	26 (7.0)	15 (4.0)	
**Total**	**444 (89.0)**	**25 (5.0)**	**30 (6.0)**		**442 (89.8)**	**32 (6.5)**	**18 (3.7)**	
**Gestational age (weeks)**								
Below 12 weeks	38 (86.4)	4 (9.1)	2 (4.6)	0.295	38 (88.4)	3 (7.0)	2 (4.6)	0.143
13 – 26	215 (88.5)	16 (6.6)	12 (5.0)		209 (86.7)	23 (9.5)	5 (3.9)	
27 – 32	163 (90.1)	4 (2.2)	14 (7.7)		165 (93.2)	5 (2.8)	7 (4.0)	
Above 33 weeks	28 (90.3)	1 (3.2)	2 (6.5)		30 (96.8)	1 (3.2)	0 (0.0)	
**Total**	**444 (89.0)**	**25 (5.0)**	**30 (6.0)**		**442 (89.8)**	**32 (6.5)**	**18 (3.7)**	

*P < 0.01, **P < 0.05, ***P < 0.001

The results have shown that willingness to return to the same facility in subsequent pregnancies for ANC services rating ranged from 77.4 to 92.1%. The average willingness to return to the same facility was 89.0%. The lowest and highest willingness to return to the same facility was at N’gombe (77.4%) and Kabwata (92.1%) clinics, respectively (Fig. 3). In terms of the sociodemographic characteristics and facility attributes, the willingness to return to the health facility was associated with participants’ place of residence (*p* < 0.05) and their educational attainment (p < 0.05) (Table 4).

Willingness to recommend the health facility to relatives or friends ranged from 71.0 to 96.8%. The lowest willingness to recommend the facility was among the respondents at N’gombe clinic (71.0%) and the highest was from Kalingalinga (96.7%) and Kanyama (96.8%) clinics. Participants’ willingness to recommend to others was statistically significant (*p* < 0.001), however, there was a statistical association with willingness to return to the same health facility (Table 4).

## Discussion

This study draws on Donabedian’s framework on assessing the quality of care [26], to investigate pregnant women’s satisfaction with the quality of antenatal care, and willingness to return or recommend it to others, in Lusaka district, Zambia.

### ‘Structural’ aspects of care

The section highlights a few structural concerns relative to the quality of ANC quality. We found that more than half of the participants were satisfied with the overall quality of the ANC received. However, the results showed that ‘travel distance to a health facility’, and ‘place of residence’ were significantly associated with participants’ satisfaction with ANC quality. We also found that pregnant women in this study were more satisfied with ‘access to health facilities’ and the ‘waiting time on admission’. Thus, participants rated ‘cleanliness of the ward’ high (58.3%) whereas ‘sanitary facilities in the ward’ received a less than average rating. Overall, the findings are consistent with the findings of previous studies in Nigeria and Mozambique. Health facility ‘cleanliness’ score in the Nigerian study was 6.23 out of 11 [33]. In Mozambique, ANC clients complained about the challenges of accessing health facilities coupled with the hours they spend at the clinic, regardless of the uncertainty of receiving ANC [34]. Although ‘physical aspects’ of care appear to be generic in many LMICs like Zambia, the level of satisfaction with the structural constructs of ANC quality does not suggest an acceptable level of ANC that can help the country achieve improved maternal outcomes [3, 22]. This is due to the fact that pregnant women may experience frustration and feelings of helplessness when confronted with obstacles such as long distances or difficult transportation when attempting to access care [25]. Similarly, when pregnant women are required to wait for extended periods of time to receive care, it may contribute to feelings of anxiety and stress [5], and could negatively affect their overall satisfaction with ANC [34]. Therefore, improving transportation options and reducing wait times can enhance the quality of ANC and lead to better health outcomes for both the pregnant women and children. Certain sociodemographic characteristics, such as a monthly household income between 3000 and 6000 Kwacha (ZWM), a gestational age of 27 weeks or more, and completion of secondary school, were significantly associated with pregnant women’s satisfaction with the physical aspects of ANC quality, according to our findings. The majority of the households of participants lived on less than 3000 kwacha and more than half had attained secondary-level education in this study. Besides this, participants whose gestation was 27 weeks or more, were 4 times more likely to be satisfied with the ‘physical aspect’ of care.

Overall, education, income and gestation consistently affect pregnant women’s assessment of the quality of ANC in low and middle income settings [9, 10, 15–17, 23]. Indeed, pregnant women who attain secondary level education and beyond may be more likely to understand the importance of the physical environment of ANC and may have high expectations about the quality of care in the nation’s capital district. In addition, participants in this study who lived on higher household incomes may have more resources available to them, such as access to transportation or the ability to travel far distances to the ANC clinics within Lusaka with the expectation of receiving better quality care. This may help explain the low satisfaction with ANC quality in this study. The results also showed that participants who had reached 27 weeks or more of gestation may have had more opportunities to interact with care providers and to receive more comprehensive care, which can contribute to higher satisfaction with ANC. These factors might help explain the low satisfaction rating of the physical environment of care at Mtendere and N’gombe ANC clinics when compared to rural clinics at Chainda. However, previous studies reported concerns related to facility logistics rather than the location. For instance, women using care were asked to provide certain basic supplies [35], and hospital size was associated with the poor physical environment of care in Ghana [36].

### ‘Process’ aspect of care

Donabedian’s framework for assessing healthcare quality classified the ‘process of care’ into two categories: ‘interpersonal’ and ‘technical’ aspects of care [24]. For the ‘interpersonal dimension of care, 57.9% of participants demonstrated ‘full satisfaction’ with ‘privacy maintained during care’, followed by ‘encouragement at delivery (56.9%)’ while slightly more than half were satisfied with ‘politeness, courtesy, and respect shown by midwives’. In a systematic review of midwives’ attitudes and behaviours during care, Mannava et al., found that rudeness, neglect, and verbal abuse, were behaviours during ANC, which contributed to low satisfaction [11]. Consequently, role stress was found to have contributed to the delay in seeking ANC among pregnant women in a busy District Hospital in Ghana [35]. In that study, 74.7% of the participants received antenatal care in urban clinics. The dissatisfaction rating for the ‘physical aspects’ of ANC quality was related to long waiting times in urban clinics at Mtendere and N’gombe in Lusaka district. The delayed access to ANC could be a result of staff workload. Previous studies have shown that staff workload may lead to stress and burnout [20, 37], due to staff shortages and increased ANC attendants [34]. Therefore, nurses’ ability to provide good quality care not be likely in this circumstance, which may explain pregnant women’s low rating of the level of quality ANC concerning the technical and interpersonal aspects of care.

Furthermore, participants who travelled for more than 30 minutes to seek care were two times more likely to be satisfied with the technical aspects of ANC quality. A related finding was reported in Tanzania, Ghana, and Burkina Faso [38]. In that study, pregnant women who travelled long distances to access care reported that they were more likely to receive comprehensive medical examinations, including blood tests, urine tests, and ultrasounds. They also reported that healthcare providers were more likely to take their health concerns seriously and provide appropriate treatment. In contrast, women who lived closer to the facility reported that ANC services were rushed and of poor quality, and that healthcare providers did not provide enough information or explain procedures clearly [38]. In an Ethiopian study, pregnant women who travelled long distances to ANC facilities reported receiving comprehensive medical examinations, including measurements of blood pressure, weight, and foetal growth [14]. In contrast, women who lived closer to the facility reported receiving inadequate information or explanation about care procedures and laboratory results [14]. The findings have shown that pregnant women who travelled long distances for care may have gone there because of the good care that they or other people received which explains why they chose the facilities in the first place. Thus, intensifying monitoring and oversight responsibility by the Ministry of Health may improve the’ process’ of ANC.

Another component of the ‘technical aspect’ of care was related to the ‘competency of care providers’ and ‘opportunity to clarify doubts about laboratory results’. These components were given the highest rating in this category. Consistent with previous studies in Nigeria and Tanzania, the competency of the healthcare provider saved the lives of pregnant women who had health problems that previous health facilities she received ANC missed [14, 19]. Pregnant women in Tanzania noted that detailed explanations about their laboratory results, appropriate counselling, and assurance of safety and treatment options, gave them some confidence and a better understanding of their health status [14]. These were their motivation for the high rating of the technical aspects of care in those studies [14, 19].

Overall, concerning the ‘distances to health facility’ and the ‘competency of providers’, it must be noted that Lusaka District is home to the highest-level tertiary healthcare in the country. Thus, as the country’s capital, it is also likely that the most qualified health professionals could be serving in those health facilities, and thus, women who utilised ANC there may have a more satisfying experience when compared with ANC in a rural clinic. From the socio-demographic characteristics, 71.3% were multiparous pregnant women. Therefore, the motivation to utilise long-distance ANC clinics may be influenced by previous experiences using nearby facilities or satisfactory experiences receiving ANC from more distant clinics. Overall, the positive attitudes towards certain clinics could reflect their more realistic expectations based on previous experiences with health facilities.

In this study, we found that participants who were at the gestation age of pregnancy of more than 27 weeks, were more likely to be satisfied with the physical, interpersonal, and technical aspects of ANC quality. Galle et al. found that pregnant women are more likely to have a better understanding of the dimensions of ANC in such a stage of gestation, which could explain our finding [8]. It also points to the fact that healthcare providers can understand pregnant women’s varied experiences with healthcare settings, the values, cultures, and healthcare aim that would potentially be met.

### ‘Outcome’ of ANC satisfaction

Pregnant women’s satisfaction is very paramount for continued use of healthcare in the same ANC clinic and also recommend the same health facilities to other pregnant women.

Based on the Donabedian’s framework [24], and consistent with the literature on ANC satisfaction, it is noted that pregnant women’s satisfaction will engender positive views, repeated visits, and subsequent recommendations to colleagues [8]. In this study, women’s average willingness to return to the same facility was very high (89.0%). The results also show that most participants in this study were younger women of 20-24 years. This suggests that participants in the study may not have adequate knowledge and experience about care processes and what to expect during care. We make this conclusion because, 72.3% of the participants indicated that they were from the communities they received ANC, and did not have to travel elsewhere for ANC. In terms of ‘willingness to return or recommend’ a health facility, N’gombe (77.4%) and Kabwata (92.1%) clinics received the lowest and highest rating, respectively. The relatively high ‘willingness to return or recommend to others’ may suggest that pregnant women were generally satisfied with the overall quality of ANC in these clinics.

However, it was found that two sociodemographic characteristics of participants - ‘place of residence’ and women’s ‘educational attainment’, were significantly associated with the ‘willingness to return’ to the same facility. These findings agree with similar studies in Sri Lanka, Zambia, and Kenya [11, 31, 39]. Women who had attained secondary or higher level education showed an increased ‘willingness to return’ to the same facility in Zambia [10, 39]. However, some deterring attitudes of healthcare providers such as ‘rudeness while providing care’, ‘inappropriate treatment from providers’, ‘lack of essential medicines at the pharmacy’, ‘poor sanitary facilities’, and unconsented and unauthorized transfer to a higher level facility for continuity of ANC among others, accounted for participants unwillingness to return or recommend to other participants [9, 10, 39]. The results of this study suggest that a lack of formal knowledge and health literacy regarding ANC may have affected women’s decisions to avoid N’temdere and N’gombe health facilities. Women’s education levels may affect their knowledge of what to expect from ANC and how it works. A study in Sri Lanka [31] found that negative midwife attitudes towards women with lower levels of education or no education at all during care were linked to a higher risk of not returning for future pregnancies. Overall, given the culturally-diverse nature of the population in urbanised areas like N’gombe, in this study, it may be helpful to increase ANC counselling sessions to explain procedures and possibly create room to accommodate clients’ values, and positive beliefs in order to satisfy pregnant women’s desires. Younger women made up the bulk of participants, and their lower rates of education and desire to “return to” (but not “recommend to others”) may suggest that they were reluctant to complain about poor treatment quality. Most of them probably didn’t have the money to travel to a more distant healthcare facility, so they could easily make believe that they did not have any other options [11].

Furthermore, it must be noted that some first-time pregnant women may choose certain health facilities based on recommendation by pregnant women [9, 10]. Consequently, providers should emphasise unilateral behaviours such pleasant reception and attitudes, offering enough health information to customers, maintaining a clean facility, etc. as essential methods to ensure ‘client satisfaction,’ ‘to return,’ and’ recommend to’ others. The results confirm and lend credence to Donabedian’s observations that the ‘structural’ and ‘process’ components of the issue are more important than the ‘outcome [24].

### Implications for policy and practice

The findings from the study highlight the need for government to invest more in the quality of antenatal healthcare by offering mentorship programs for care providers focused on the identified issues across the three dimensions of care (interpersonal, technical, and physical aspects). Strengthening these factors will immensely affect women’s perception of the quality of prenatal care in clinics. The identified factors associated with maternal satisfaction provide valuable information for improvements in perinatal care in Lusaka district public clinics. Although women were willing to recommend antenatal care at these facilities to relatives and friends, this did not reflect their satisfaction with the care received. Participants’ concerns could be addressed by intensifying periodic feedback by policymakers and hospital managers as part of healthcare quality assurance processes.

We recommend that the health facilities should be equipped with essential equipment and consumables and ensure provider adherence to international and national antenatal care protocols and service delivery guidelines. Additional research and programming are needed to strengthen health systems to provide acceptable quality care -with the principles of respectful and dignified maternity care at the core of service delivery - to realize every woman’s right to respectful care and to improve maternal health outcomes. Going forward, a nationwide assessment of specific aspects of pregnant women’s experiences with ANC will be useful to identify areas of dissatisfaction in maternal care provision for improvement.

### Limitations of the study

The study has some limitations. Despite the high response rate, some selection bias could not be avoided as women without any access to antenatal care were not reached. In addition, women younger than 18 years old were excluded from the study for ethical reasons. Parental permission was required which could, could compromise participants’ privacy and anonymity. Pregnant women in emergency wards were also excluded. Thus, our findings may be only applicable to perinatal care of low-risk women. However, pregnant women were included irrespective of the number of antenatal clinic visits; this may limit interpretation because some women may not have had enough exposure to the clinic to enable them to make concrete judgments on perception and satisfaction.

Despite these limitations, LH and the field assistants were trained to gather responses ethically from the respondents. The tool used in collecting the data was pre-tested and used in a previous study in Zambia, and subsequently programmed into Google forms which ensured that quality was maintained by including appropriate skip logic and ensured that no questions were skipped without getting a response, hence ensuring that there was no missing data. Privacy was maintained by conducting the interviews in a separate room independent of healthcare providers and with adequate assurance of confidentiality of information.

## Conclusion

In line with Donabedian’s proposition on quality of care and its implication on care continuity and health outcome, the level of satisfaction with ANC in this study was relatively low to achieve the full life-saving potential that antenatal care could provide as found elsewhere. The study identified several facility-based practices that negatively impact pregnant women’s satisfaction with ANC that may be alterable by health managers and care providers such as lack of sanitary facilities, beds, medicines, courtesy, politeness or respect from the midwives, and long waiting time. We recommend qualitative exploration into the exact forms of behaviours and attitudes of ANC providers pregnant women receive during ANC in the study area. This will help policy initiatives that will engender increases in health facility ANC service utilisation in Zambia.

### Supplementary Information

**Additional file 1:** Supplemental file 1. Appendix 1. Survey tool: (questionnaire).

## Data Availability

The datasets used and/or analysed during the current study is available from the corresponding author on reasonable request.

## Abbreviations

ANC: Antenatal care
MMR: Maternal Mortality Rate
MoH: Ministry of Health
SSA: Sub-Saharan Africa
UNICEF: United Nations International Children’s Emergency Fund
UWCBMREC: University of the Western Cape Biomedical Research Ethics Committee
UNZABREC: University of Zambia Biomedical Research Ethics Committee
WCBA: Women of Child-Bearing Age
WHO: World Health Organization
ZDHS: Zambia Demographic Health Survey
