# Supplementary material for: Pregnant women’s satisfaction with the quality of antenatal care and the continued willingness to use health facility care in Lusaka district, Zambia
Source: BMC Pregnancy Childbirth. 2024 Jan 2;24:20. doi: 10.1186/s12884-023-06181-5 (PMC10759641; doi:10.1186/s12884-023-06181-5)
Supplement: Supplementary file 1 — Additional file 1: Supplemental file 1. Appendix 1. Survey tool: (questionnaire). [file 12884_2023_6181_MOESM1_ESM.docx]

**Supplemental file 1**

##### : Survey tool: (questionnaire)

**Section A: Characteristics of the study population**

1. What is your age?
2. What is your marital status: 1. Single 2. Married 3. Divorced 4. Living with a stable partner 5. Other (specify)
3. Specify marital status if “Other” is selected
4. How old is your pregnancy (in weeks)?
5. How many children who are alive do you have?
6. How many times have you given birth to a child with a pregnancy of 6 months old or above (Parity)?
7. What is your occupation – 1. Employed 2. Not Employed 3. Other (specify)
   1. What kind of work do you do? (if respondent answers “employed” above)
   2. If other, what does that mean?
8. What does your husband do for a living? 1. Employed 2. Not Employed 3. Other (specify)
   1. What kind of work does he do? (if respondent answers “employed” above)
   2. If other, what does that mean?
9. How long does it take you to reach this clinic/hospital?
10. Is this the nearest clinic that offers ANC for you? 1. Yes 2. No 3. I don’t know
    1. (If respondent selects no) Which one is the nearest clinic to you that offer the same services you have come for at this clinic?
    2. Why did you choose to be coming to this clinic and not the nearest clinic you just mentioned above?
11. Household estimated monthly income (in Zambian kwacha) – 1. <=ZMW1000 2.>1000 - <=3000 3. >3000 - <=6000 4. >6000
12. What is your ethnic group?
13. What is your highest level of education completed – 1. None 2. Primary 3. Secondary 4. Diploma 5. Degree 6. Above degree (Postgraduate degree) 7. Other (specify)
    1. Please ask the respondent to specify if they selected other
14. What is your town of residence

**Section B: Levels of Satisfaction**

1. Where you satisfied or dissatisfied with the items below: On a scale of between 1 to 5 with 1 being fully dissatisfied and 5 being very satisfied, how much where you satisfied (or dissatisfied) with the items below:
   1. Fully dissatisfied
   2. Somewhat dissatisfied
   3. Neither satisfied nor dissatisfied
   4. Somewhat satisfied
   5. Fully satisfied

| **How satisfied (or dissatisfied) are you with?** | Fully dissatisfied | Somewhat dissatisfied | Neither satisfied nor dissatisfied | Somewhat satisfied | Fully satisfied |
| --- | --- | --- | --- | --- | --- |
| Ability to reach the hospital from your residence |  |  |  |  |  |
| Waiting time since arrival at the hospital until you were first registered in the ward |  |  |  |  |  |
| Privacy maintained by the health staff during the care |  |  |  |  |  |
| Encouragement at delivery by the health staff |  |  |  |  |  |
| Politeness, courtesy and respect shown by doctors |  |  |  |  |  |
| Politeness, courtesy and respect shown by nurses |  |  |  |  |  |
| Politeness, courtesy and respect shown by midwives |  |  |  |  |  |
| Medical facilities in the ward (drugs, equipment, etc.) |  |  |  |  |  |
| Competency of the hospital health staff in providing care to both you and your baby |  |  |  |  |  |
| Health advices given by the hospital health staff to look after the baby |  |  |  |  |  |
| Opportunity given to you to clarify doubts about the care of the newborn |  |  |  |  |  |
| Opportunity given to you to clarify doubts about the laboratory test results |  |  |  |  |  |
| Cleanliness in the ward |  |  |  |  |  |
| Availability of beds in the ward |  |  |  |  |  |
| Sanitary facilities (water, toilets, bathrooms) in the ward |  |  |  |  |  |

**Source:** (Senarath, Fernando and Rodrigo, 2006)

**Section C: Quality of Services**

1. How do you rate the quality of the services below?

| **Perception of quality level** | **Excellent** | **Good** | **Poor** | **Very Poor** |
| --- | --- | --- | --- | --- |
| Clinic |  |  |  |  |
| Attending doctor |  |  |  |  |
| Attending nurse/midwife |  |  |  |  |
| Laboratory services |  |  |  |  |
| Pharmacy support |  |  |  |  |

**Section D:** **Causes of satisfaction with antenatal care**

1. Kindly confirm true or false your satisfaction with the following aspects of antenatal care

| **Causes of satisfaction with antenatal care** | True | False |
| --- | --- | --- |
| Reception by health staff is good |  |  |
| Clear instructions given by the health providers |  |  |
| Medical orderly available with doctor in ANC |  |  |
| Good, clean clinic |  |  |
| Good clinic arrangement |  |  |
| Good laboratory services |  |  |
| Satisfied with services of the clinic |  |  |
| Timely work |  |  |

**Section E: Causes of dissatisfaction among pregnant women**

1. In your opinion, state whether the following items below are a cause of dissatisfaction to you.

| **Causes of dissatisfaction among pregnant women** | True | False |
| --- | --- | --- |
| Laboratory services |  |  |
| Crowding clinic in the morning |  |  |
| Non-availability of Arabic speaking doctor |  |  |
| Health education services not good |  |  |
| No explanation of antenatal clinic results |  |  |
| Not listening to complaints of pregnant women |  |  |
| Unavailability to see the doctor |  |  |
| Long waiting time |  |  |
| Disagree with some test results |  |  |
| Not consulted on my opinion regarding actions being taken on the pregnancy i.e., natural or caesarean section |  |  |

**Section F: Willingness to Return**

1. If you get pregnant again would you come back to this clinic for ANC check-ups? – 1. Yes 2. No 3. Maybe
   1. If yes to the above, why would you return?
   2. If no to the above, why would you not return?

**Section G: Recommendation**

1. Would you recommend this clinic to a relative or friend for their antenatal check-ups? – 1. Yes 2. No 3. Don’t know
   1. If yes to the above, why would recommend this clinic for ANC services?
   2. If no to the above, why would you not recommend this clinic for ANC services?

**Appendix II: Translated Questionnaire into Nyanja**

**MAKHALIDWE A ANTHU OWERENGERA**

1. Zaka zanu ndi zingati
2. Kodi banja lanu ndi lotani? 1. Osakwatiwa 2. Okwatiliwa 3. Olekana 4. Kukhala ndi mnzanu wokhazikika
3. Mgwirizano
4. Nambala ya bana balimoyo
5. Nchito
6. Ndalama zomene mugwira pa mwedzi (Mu kwacha) – 1. <=ZMW1000 2. >1000 - <=3000 3. >3000 -<=6000 4. >6000
7. Nchito ya amuna anu
8. Mtundu
9. Mulingo wamaphunzilo yanu 1. Kulibe 2. Primary 3. Secondary 4. Diploma 5. Degree 6. Above degree (Postgraduate degree)
10. Mzinda
11. Kutalimpa kwa mimba

**Section B**

1. Ndimwe okondwela nangu osakondwe pali vili pa nyansi?
2. Pa skelo yama nambala yoyambila pa 1 kufika pa 5, elo 1 yilangiza kusakondwe kwambiri na 5 yilangiza kukondwera kwa mbiri, mwenze okondwera bwanji olo osakondwera pali vintu votantikiwa panyansi?
   1. Osakondwera maningi
   2. Osakondwera pangono
   3. Siniziwa bwino
   4. Okondwera maningi
   5. Okondwera pangono

| **Ndimwe okondwela nangu osakondwe pali vili pa nyansi?** | Osakondwera maningi | Osakondwera pangono | Siniziwa bwino | Okondwera maningi | Okondwera pangono |
| --- | --- | --- | --- | --- | --- |
| Mphavu zofikira kuchipatala kuchokera komwe mumakhala |  |  |  |  |  |
| Nthawi yomwe mumakhala kuyambira pomwe mwafika kuchipatala mpaka pomwe bazamipasani m'chipinda chogona |  |  |  |  |  |
| Zachinsinsi zimasungidwa ndi azachipatala posamalira |  |  |  |  |  |
| Chilimbikitso pakubereka kwa azachipatala |  |  |  |  |  |
| Aulemu, ulemu ndi ulemu womwe akuwonetsa madotolo |  |  |  |  |  |
| Ulemu, ulemu ndi ulemu zosonyezedwa ndi anamwino |  |  |  |  |  |
| Ulemu, ulemu ndi ulemu zosonyeza azamba |  |  |  |  |  |
| Malo azachipatala mu wadi (mankhwala osokoneza bongo, ndi zina zambiri) |  |  |  |  |  |
| Kuchita bwino kwa ogwira ntchito kuchipatala posamalira inu ndi mwana wanu |  |  |  |  |  |
| Malangizo azaumoyo operekedwa ndi ogwira ntchito kuchipatala kuti aziyang'anira mwanayo |  |  |  |  |  |
| Mwayi wopatsidwa kwa inu kuti ufotokoze kukayikira za chisamaliro cha mwana wakhanda |  |  |  |  |  |
| Mwayi womwe mwapatsidwa kuti mufotokozere kukayika pazotsatira za labotale |  |  |  |  |  |
| Ukhondo mu mzinda wa chogonera |  |  |  |  |  |
| Kupezeka kwa mabedi mu mzinda |  |  |  |  |  |
| Malo yamuchipatala (madzi, zimbudzi, mabafa) mu ward |  |  |  |  |  |
| Umoyo wanu |  |  |  |  |  |

**Source:** (Senarath, Fernando and Rodrigo, 2006)

1. Kodi mumawerengera bwanji ntchito zomwe zili pansipa?

| **Kuzindikira kwa mulingo wabwino** | **Zabwino kwambiri** | **Zabwino** | **Osauka** | **Osauka Kwambiri** |
| --- | --- | --- | --- | --- |
| Clinic |  |  |  |  |
| Kupita kwa dokotala |  |  |  |  |
| Kupita kwa namwino / mzamba |  |  |  |  |
| Nthawi yakudikirira |  |  |  |  |
| Ntchito zantchito |  |  |  |  |
| Thandizo la Pharmacy |  |  |  |  |

1. Zomwe zimakhutiritsa chisamaliro chakubereka

| ZOMWE ZIMAKHUTIRITSA CHISAMALIRO CHAKUBEREKA | ZOWONA | ZABODZA |
| --- | --- | --- |
| Malangizo omveka bwino operekedwa ndi othandizira azaumoyo |  |  |
| Malangizo omveka bwino operekedwa ndi othandizira azaumoyo |  |  |
| Zachipatala mwadongosolo amapezeka ndi adokotala ku ANC |  |  |
| Chipatala chabwino, choyera |  |  |
| Kukonzekera bwino kwa chipatala |  |  |
| Ntchito zabwino zasayansi |  |  |
| Okondwera ndi ntchito |  |  |
| Ntchito yake panthawi yake |  |  |

1. Mukuganiza kwanu, fotokozerani ngati zinthu zotsatirazi sizikusangalatsani

| **ZOMWE ZIMAYAMBITSA KUSAKHUTAIRA PAKATI PA AMAYI APAKAATI** | **ZOWONA** | **ZABODZA** |
| --- | --- | --- |
| Ntchito zantchito |  |  |
| Kuchulukana kuchipatala m'mawa |  |  |
| Kusapezeka kwa dokotala wolankhula Chiarabu |  |  |
| Ntchito zophunzitsa zaumoyo sizabwino |  |  |
| Palibe kufotokozera za zotsatira zakuchipatala |  |  |
| Osamvera madandaulo a amayi apakati |  |  |
| Kulephera kuwona dokotala |  |  |
| Nthawi yayitali yoyembekezera |  |  |
| Sindikugwirizana ndi zotsatira zina za mayeso |  |  |
| Osandifunsa malingaliro anga okhudza zomwe zikuchitika pathupi monga, gawo lachilengedwe kapena la opaleshoni |  |  |

1. Nthawi yo mukakhala ndi pakati nafuti, mungabwerere ku chipatala ichi?

1. Inde 2. Awe 3. Siniziwa

1. Kodi mungalangize za chipatala ichi kwa wachibale kapena mnzanu kuti akawone ngati ali ndi pakati?

1. Inde 2. Awe 3. Siniziwa

1. kodi ndinu okondwera ndi ANC yomwe mwalandira kuchipatala pano ? –
   - 1. Okondwera Maningi 2. Okondwera 3. Osakondwera
